# Tissue segmentation‐based electron density mapping for MR‐only radiotherapy treatment planning of brain using conventional T1‐weighted MR images

**DOI:** 10.1002/acm2.12654

**Published:** 2019-07-01

**Authors:** Huan Yu, Michael Oliver, Konrad Leszczynski, Young Lee, Irene Karam, Arjun Sahgal

**Affiliations:** ^1^ Department of Medical Physics, Northeast Cancer Centre, Health Sciences North, Medical Sciences Division, Northern Ontario School of Medicine, Faculty of Medicine Laurentian University, Lakehead University Sudbury ON Canada; ^2^ Department of Medical Physics, Odette Cancer Centre Sunnybrook Health Science Center Toronto ON Canada; ^3^ Department of Radiation Oncology University of Toronto Toronto ON Canada; ^4^ Department of Radiation Oncology, Odette Cancer Centre Sunnybrook Health Science Center Toronto ON Canada

**Keywords:** brain, MR pseudo CT, MR synthetic CT, MR‐linac, MR‐only treatment planning

## Abstract

**Purpose:**

Magnetic resonance imaging (MRI) is the primary modality for targeting brain tumo**rs** in radiotherapy treatment planning (RTP). MRI is not directly used for dose calculation since image voxel intensities of MRI are not associated with EDs of tissues as those of computed tomography (CT). The purpose of the present study is to develop and evaluate a tissue segmentation‐based method to generate a synthetic‐CT (sCT) by mapping EDs to corresponding tissues using only T1‐weighted MR images for MR‐only RTP.

**Methods:**

Air regions were contoured in several slices. Then, air, bone, brain, cerebrospinal fluid (CSF), and other soft tissues were automatically segmented with an in‐house algorithm based on edge detection and anatomical information and relative intensity distribution. The intensities of voxels in each segmented tissue were mapped into their CT number range to generate a sCT. Twenty‐five stereotactic radiosurgery and stereotactic ablative radiotherapy patients’ T1‐weighted MRI and coregistered CT images from two centers were retrospectively evaluated. The CT was used as ground truth. Distances between bone contours of the external skull of sCT and CT were measured. The mean error (ME) and mean absolute error (MAE) of electron density represented by standardized CT number was calculated in HU.

**Results:**

The average distance between the contour of the external skull in sCT and the contour in coregistered CT is 1.0 ± 0.2 mm (mean ± 1SD). The ME and MAE differences for air, soft tissue and whole body voxels within external body contours are −4 HU/24 HU, 2 HU/26 HU, and −2 HU/125 HU, respectively.

**Conclusions:**

A MR‐sCT generation technique was developed based on tissue segmentation and voxel‐based tissue ED mapping. The generated sCT is comparable to real CT in terms of anatomical position of tissues and similarity to the ED assignment. This method provides a feasible method to generate sCT for MR‐only radiotherapy treatment planning.

## 1 Introduction

Magnetic resonance imaging (MRI) is the modality of choice for defining brain tumor volume in precision radiotherapy techniques such as stereotactic radiosurgery (SRS) and stereotactic ablative radiotherapy (SABR) because MRI provides high soft‐tissue contrast, functional information, and high resolution, which is superior to what can be provided by a planning CT. However, MRI alone is not sufficient in radiotherapy treatment planning (RTP) due to its lack of electron density (ED) information in its image voxel intensity values for radiation dose calculation. Conventionally, the radiation dose has to be calculated using CT images which are registered with MR images. This image registration process will introduce errors in defined tumor volumes.^1^ Although these errors are usually small due to the rigid structure of the skull, image registration errors can be significant in some cases when MR and CT scans have differences in positions or setup.^2^ This may lead to geometrical miss of target volumes as the target volumes are usually small and increase in dose to nearby critical organs. This, in turn, would compromise the effectiveness of treatment and the patient’s quality of life. MR‐only treatment planning will eliminate image registration error, minimize patient setup error, and reduce unnecessary radiation to patients from multiple CT scans.

One of the major benefits of MR‐only RTP is improved workflow and reduced burden on the patient in terms of multiple visits/scans. Also, MR‐only RTP is what will be needed in the future for new technologies such as the MR‐Linac.

One solution to the problem is to derive ED information from MR images to generate synthetic CT (sCT) images. It is not a trivial problem since, in commonly used MR images, air and cortical bone have no difference in MR image voxel intensity values. Some tissues have a similar or overlapping MR intensities and very different EDs, for example, bone, eye, cerebrospinal fluid (CSF), brain, trabecular bone, muscle and fat tissues. The MR image voxel intensity values are not as standardized as those of CT and change with scanning parameters, MR scanner manufacturer, magnetic field strengths, and patients. It poses a big challenge to separate tissues based on the absolute voxel intensity values of MR images. Many postscan MR voxel intensity standardization algorithms such as those based on tissue segmentation were developed.^3^ In literature, atlas‐based deformable image registration, MR bone imaging‐based tissue classification or segmentation, and machine learning‐based convolutional neural network (CNN) are the main approaches used to generate MR‐based sCT.^3^, ^4^, ^5^, ^6^, ^7^, ^8^, ^9^, ^10^, ^11^, ^12^, ^13^, ^14^, ^15^, ^16^

Atlas‐based approaches usually applied deformable registration algorithms to a pair of population based coregistered MR/CT images from one or multiple atlases to match a new patient’s MR images. The voxels of the patient’s MR images were assigned ED values by warping electron densities from the deformed CT images.^3^, ^5^, ^6^ However, the uncertainty of image registration increases with the variation of a patient’s anatomy and pathology with respect to the atlases. Some patients were excluded from these studies due to significant anatomic or pathologic variations. The deformable registration error can be as much as 3 mm reported by a study evaluating some deformable registration methods.^4^

Several approaches utilized a specialized MR sequence such as ultra‐short echo time (UTE) to differentiate air and bone.^7^, ^8^, ^9^, ^10^, ^11^ These sequences typically have a low signal to noise ratio and tissue contrast, bone, and other soft tissues such as fat and muscle cannot be clearly differentiated. Therefore, typical bone scan sequences in these studies included multiple UTE, T1, and T2 sequences. Some postscan image processing methods were developed to process multiple MR image series obtained from the bone scan sequences to classify image voxels as bone, air, and other soft tissues. Then, either bulk or voxel‐based ED assignment was used to generate sCTs.^9^, ^10^, ^11^, ^12^, ^13^ It is a costly approach. Errors due to MR artifacts such as chemical shift and motion in any of these MR sequences can be inherited by the generated sCT.

Deep learning‐based CNN techniques used a trained convolutional kernel to predict the ED of each voxel of a patient’s MR images to generate sCT.^15^, ^16^ The kernel consists of many layers of small image filters associated with many parameters trained using coregistered MR and CT image datasets. These methods used only one set of T1‐weighted MR images and did not need deformable registration. High‐resolution MR images were required since small sizes of filters were used in the kernel to avoid blurring. Therefore, these methods require computers with high computing power such as one with GPUs and large random access memory (RAM) for training kernels and generating sCTs. Large number of high‐quality training data are essential for this approach as any error introduced into kernel can produce inherited errors into generated sCTs.

In our previous study,^17^ a semiautomated method was developed which can segment cortical bone from T1‐weighted MR and generate “CT‐like” MR for radiation treatment image verification by identifying air region (sinuses and air way) through several slices of manual contours. The generated MR‐based digitally reconstructed radiographs (DRR) have been verified for geometric accuracy of segmented bone.

However, to generate sCT for dose calculation, electron densities of soft tissues have to be accurately assigned, especially for those soft tissues such as brain that may have MR intensities overlapping with those of trabecular bone and fat. Contrast enhanced T1‐weighted MR images clinically used have high contrast (high intensity value) where a tumor is located resulting in contrast‐related artifacts. Cerebrospinal fluid usually has low MR intensity values which may partially overlap with those of bones.

In MR neuroimaging, methods have been developed that can automatically segment brain tissues using conventional MR sequences such as T1‐weighted images.^18^, ^19^ These methods can segment brain tissue without using atlas and deformable registration. Moreover, these methods are not very sensitive to MR voxel intensity variation due to anatomical variation or scan parameters since the methods do not rely on the thresholding of standardized voxel intensity values of MR images obtained from a set of training data. The MR voxel intensity range of brain for an individual patient was found by searching representative voxels of brain tissue with certain anatomic or textural properties in the patient’s MR images.

In this study, a voxel‐ based ED assignment method has been developed by segmenting tissues: air, brain, CSF, cortical bone, trabecular bone, eyes, muscles and fat from a patient’s MR images. A sCT was generated by assigning voxel‐based electron density values to corresponding types of tissue’s voxels.

## 2 Materials and methods

### 2.A Tissue segmentation

**Figure 1:**
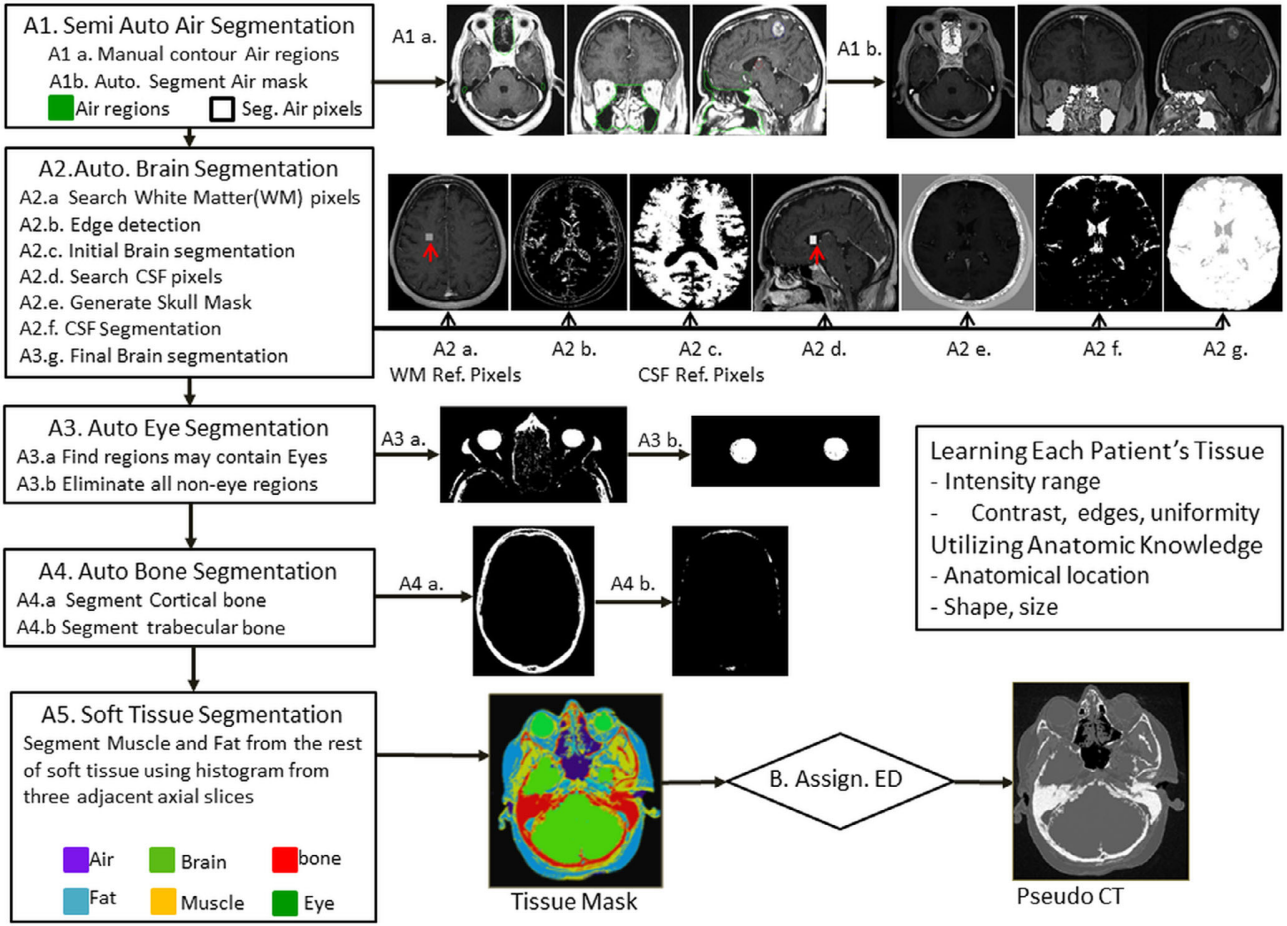
Systematic of tissue segmentation‐based electron density mapping method

This process was used to separate tissues with similar intensities in T1‐weighted MR images and a large difference in electron density such as cortical bone, trabecular bone, brain, CSF, eyes, muscle and fat. Tissues were automatically segmented by utilizing information such as high soft‐tissue contrast, edge detection, anatomical location, shapes, and statistical features of the tissue such as mean (μ), standard deviation (σ), and uniformity (Fig. 1).

The uniformity of a tissue *U*_tissue_ within a defined window such as 1 cm × 1 cm is calculated as

(1) $$U_{\text{tissue}}=\mu (p_{i})/\sigma (p_{i})$$

where voxel $p_{i}\in$ voxels within the sample window of a tissue.

#### 2.A.1 Air mask generation

As described in our previous study,^17^ separating air from cortical bone was achieved by manually contouring air regions enclosing sinuses and airway in less than 12 slices. The entire air regions in the head then were obtained by interpolating these contoured slices to slices in between. This is the only manual step in this process. Afterward, an in‐house algorithm developed with IDL8.7 (ITT Visual Information Solutions) calculated the statistic mean (μ_Air_) and standard deviation (*σ*Air) of MR intensity values of the air, then generated an air mask by subtracting soft tissues enclosed in the air regions. Subsequently, it will generate other tissue segmentation as follows.

#### 2.A.2 Automated brain and CSF segmentation

##### Determination of statistical intensity range of brain

First, search a voxel with the largest intensity uniformity calculated using eq. (1) within a defined 1cm x 1cm window centered on the voxel in the middle coronal slice. The mean intensity of voxels within the window centered on the voxel is the reference intensity of brain (wmIntensity). The statistic brain tissue intensity range is from $\left[0.53\times \text{wmIntensity},1.35\times \text{wmIntensity}\right]$.^15^

##### Edge detection

MR images were processed by thresholding using intensity range found in Section “Determination of statistical intensity range of brain” to separate the skull from brain tissue. To enhance edge detection, all voxel intensities out of this range were set to 0. Then, Canny edge detection algorithm^20^ was applied to each slice in axial and sagittal directions. The largest external contours of these edges within the skull for each slice were identified for brain segmentation. The broken edges were enhanced by searching the external path of the edges and closing any gaps more than one voxel distance between two closest voxels of the edge by adding missing voxels into the edges along the shortest path between these edge voxels. All the edge voxels were set to maximum intensity values to prevent leakage when using region growing algorithm.

##### Initial brain segmentation

Firstly, brain mainly consisting of white matter with high uniformity was segmented by applying region growing algorithm from the reference voxel of white matter in three dimensions with intensity range $\left[0.7\times \text{wmIntensity},1.2\times \text{wmIntensity}\right]$.

##### Determine statistical intensity range of CSF

Cerebrospinal fluid reference voxels were searched within an initial segmented brain in a window size of 5 mm × 5 mm × 5 mm with a maximum uniformity and intensity less than 0.53 times of wmIntensity. The mean (μ_CSF_) and standard deviation (σ_CSF_) of the voxels within the window were calculated.

##### Skull Mask

Due to surgery on the skull, segmented brain volume may spread into the spongy bone of the skull or soft tissue outside of the skull with a region growing algorithm.

Reference voxels of the skull were obtained by thresholding with an intensity range $\left[0,\mu_{\text{AIR}}+3\times \sigma_{\text{AIR}}\right]$ from a region within 1 cm from external body contour. A skull mask was generated by region growing algorithm from the skull reference voxels with a range $\left[0,\mu_{\text{CSF}}+2\times \sigma_{\text{CSF}}\right]$ in regions external to the segmented brain and CSF, and the edges of the brain identified in Section “Edge detection”. All the soft tissues voxels external to the skull relative to that brainin distance were added into the skull mask.

##### CSF segmentation

CSF voxels were segmented using region growing algorithm starting from CSF reference voxels which were found in Section “Determine statistical intensity range of CSF,” in brain, around brain, and spinal cord with a range from $\left[\mu_{\text{CSF}}-2\times \sigma_{\text{CSF}},\mu_{\text{CSF}}+2\times \sigma_{\text{CSF}}\right]$ constrained by the skull mask.

##### Final brain segmentation

In this final step, the brain voxels (e.g., gray matter) within groves among initial segmented brain in Section “Initial brain segmentation” were segmented when these voxels were connected with the segmented brain tissue with intensity range found in Section “Determination of statistical intensity range of brain” and did not fall within the skull mask.

#### 2.A.3 Automated eye segmentation

Eyes have well‐defined boundaries in T1‐ weighted MR images and their MR intensities are lower than those of brain. Left and right eyes are located at the anterior hemisphere relative to the brain and are symmetrical to the middle line of brain. Therefore, the eye segmentation was designed using these intensity and anatomical information. First, a brain slice with the maximum anterior posterior width was identified. In an image box which is 5 cm superior and 7 cm inferior to that brain slice at the anterior hemisphere of the head, connected regions with voxel intensity values less than 0.7 times of typical brain intensity wmIntensity were separated. From these regions, any region which was connected to the cross sagittal midline of brain, or had a too small volume (less than half of a typical eye size: 4 cm in diameter), or too large volume (more than two times of a typical eye’s size) or not a spherical shape (defined as when the maximum difference of diameters in three dimensions was more than 1 cm), were excluded from eye regions. Regions which were satisfied all above criteria were identified as left or right eye.

#### 2.A.4 Automated bone segmentation

As described in our previous study,^17^ bone mask was obtained by using region growing algorithm with similar intensity ranges as that of air. Bone segmentation in this study mainly focused on trabecular bone because the volume of trabecular bone can be quite large for some patients due to aging and pathological effect. Furthermore, the range of intensities of trabecular bone in T1‐weighted MR images overlaps with brain tissue and fat. Trabecular bone volumes enclosed in cortical bone mask were first separated as reference trabecular bone voxels. Mean (μ_trabecular_) and standard deviation (σ_trabecular_) of the MR intensities were calculated from the trabecular bone voxels. Trabecular bones such as those in spinal bone may not be entirely enclosed by a cortical bone mask due to “thinness” of cortical bone, thickness of image slices, and partial volume effect. These trabecular bone volumes were recovered by locally searching voxels in a small neighborhood window near cortical bone voxels with intensities falling within the interval $\left[\mu_{\text{spongy}}-2\times \sigma_{\text{spongy}},\mu_{\text{spongy}}+2\times \sigma_{\text{spongy}}\right]$.

#### 2.A.5 Other soft tissue segmentation

Other soft tissues, excluding brain, CSF, bone, and air cavities mainly consist of fat and muscle. Fat usually has high intensity value in T1‐weighted MR images, but relatively low electron density compared to that of muscle. The minimum (min_soft_ *)*, mean (μ_soft_), and standard deviation (σ_soft_) of soft tissues were calculated excluding bone and air. To cope with intensity variation due to the nonuniform magnetic field across the volume, μ_soft_ and σ_soft_ were calculated for each axial slice by including all soft tissue voxels from two adjacent slices to the slice. Muscle intensity was assumed to fall within the an intensity range $\left[\min_{\text{soft}},\mu_{\text{soft}}\right]$. Fat was segmented with an intensity range $\left[\mu_{\text{sof}t},\mu_{\text{soft}}+2\times \sigma_{\text{sof}t}\right]$. Contrast artifacts were obtained by thresholding voxels with an intensity range larger than $\mu_{\text{soft}}+2\times \sigma_{\text{sof}t}$.

### 2.B Generation of synthetic‐CT

Synthetic‐CT was generated by substituting the MR intensity value of each voxel of a tissue with a corresponding CT number in Hounsfield unit (HU), which has a standardized relationship of electron density of a tissue relatively to that of water.^21^ Intensities of all voxels in air mask and external contour outside of a patient’s body were mapped into a single CT number —1000 HU. MR intensity of each voxel of segmented bone and other soft tissues were mapped to a CT number within the corresponding statistical CT number range of the tissue as shown in Table 1.

**Table 1:** Description of mapping of tissue type from T1‐Weighted MR intensity range to CT

Tissue type	MR intensity range	Voxel ED mapping transform	CT (HU)
Air	All intensities	Bulk assignment	−1000
Cortical bone	μ_cbone_ ± 2σ_cbone_	Inverse linear	[700,1100]
Trabecular bone	μ_sbone_ ± 2σ_sbone_	Inverse linear	[500,700]
Brain	μ_brain_ ± 2σ_brain_	Linear	[40,75]
CSF	All intensities	Bulk assignment	45
Muscle	Min_soft tissue_, μ_soft tissue + _σ_soft tissue_	Inverse linear	[20,80]
Fat	μ_soft tissue + _σ_soft tissue, _μ_soft tissue_ + 2σ_soft tissue_	Inverse linear	[−70, −20]
Contrast	>μ_soft tissue_ + 2σ_soft tissue_	Bulk assignment	0

CSF, cerebrospinal fluid; CT, computed tomography; MR, magnetic resonance.

### 2.C Validation materials

To validate the accuracy of the generated sCT images in the head, images sets from 25 patients with brain tumor undergoing SRS or SABR were selected. This study received local Research Ethics Board approval from two institutes. Twenty patients undergoing SRS and five patients undergoing SABR had both MR and CT scans for planning. For MR scans, the patients were not immobilized in treatment position, but positioned with the head in a comfortable position from the top of the head, inferiorly to the base of the skull. The MR scans were acquired using two 1.5 T Signa HDxt MR scanners (GE Healthcare, Waukesha, WI, USA) from two institutes with intravenous Gadovist contrast (Bayer Healthcare, Monheim, Germany). In 20 SRS cases, 116 axial slices with a thickness of 1.5mm were acquired. The scan sequence was a 3D T1 FSPGR, with repetition/echo time 8.548/ 4.2 ms, number of averages 1, frequency flip angle 20, acquisition matrix 270 × 270, and voxel size 0.43 mm × 0.43 mm × 1.5 mm^3^. In five SABR cases, 62–68 axial slices with a thickness of 2.5 mm were acquired, The scan sequence was a 2D T1 FSPSE with repetition/echo time 566.7/13.3 ms, number of average 2, frequency flip angle 90, acquisition matrix 480 × 240, and voxel size 0.47 × 0.47 mm × 2.5 mm^3^, The geometrical distortion of MR images was corrected using the vendor’s algorithm. CT‐simulation images of 20 SRS were acquired using a 16‐slice Philips Brilliance CT scanner (Philips Medical Systems, Cleveland, OH, USA) with voxel size 1 × 1 × 1 mm^3^ and a stereotactic frame fixated to the skull or an Aktina fixation system for noninvasive cranial LINAC‐based SRS. CT images of five SABR were acquired using a GE lightspeed CT scanner (GE Medical Systems) with voxel size 0.74 × 0.74 × 2.5 mm^3^ with the patient immobilized in a thermal plastic mask.MR image data was registered with CT images in the Pinnacle^3^ treatment planning system v9.8 (Philips Medical Systems, Cleveland, OH, USA) using a 6 degrees of freedom, rigid‐body, mutual information algorithm. Small manual adjustments were made to match the outermost edges of the skull. MR and CT images of these patients were selected because the uncertainty of cranial MR and CT image registration has been quantified to be within 2 mm for various registration algorithms by a multi‐institutional benchmark test.^2^ The geometric distance difference between T1‐Weighted MR and CT images was measured to be less than 0.5% in the Radionics SRS xKnife planning system (Integra Life Sciences, Plainsboro, NJ, USA) and ERGO ++ (Elekta) radiosurgery planning system for the SRS cases.

### 2.D Evaluation methods

The generated sCT was compared with coregistered CT to evaluate its geometric similarity and accuracy of ED assignment by using the following criteria:

#### 2.D.1 Distances between bone contours of sCT and CT

On three axial, coronal, and sagittal slices with the largest width of external bone contours, for each voxel on the external bone contours of sCT, automatically searching for a voxel on the external bone contour of CT with a shortest distance. The shortest distance is regarded as one measurement of distance between voxels on bone contours of sCT and CT as shown in Fig. 3. The average of the shortest distances of all voxels on the evaluated external bone contours are recorded as the average distance difference of bone contours between sCT and CT.

#### 2.D.2 Dice similarity coefficient of bone and brain

The bone volumes were generated using a thresholding 200 HU respectively for both CT and sCT. The brain segmentation was evaluatedusing manual segmentation by a radiation oncologist for five patients in MR images. The Dice Similarity Coefficient (DSC) is calculated as.

(2) $${\text{DSC}}_{\text{Tissue}}=\frac{2\times \left({\text{Tissue}}_{\text{CT}}\;\cap \;{\text{Tissue}}_{\text{sct}}\right)}{{\text{Tissue}}_{\text{CT}}+{\text{Tissue}}_{\text{sct}}}$$

The mean error (ME) of electron density represented by standardized CT number — HU and the mean absolute error (MAE) of electron density error for a given segmented tissue voxels (N) was calculated using following equations.

(3) $${\text{ME}}_{\text{tissue}}=\left(\frac{1}{N_{\text{tissue}}}\right)\sum_{i=1}^{N}\left({\text{CT}}_{i}-{\text{sCT}}_{i}\right)$$

(4) $${\text{MAE}}_{\text{tissue}}=\left(\frac{1}{N_{\text{Tissue}}}\right)\sum_{i=1}^{N}\left|{\text{CT}}_{i}-{\text{sCT}}_{i}\right|$$

The volumes of soft tissues came from MR tissue segmentations of sCTs within the external body contours of corresponding CTs. The external contours were obtained with a thresholding of — 400 HU. Due to significant differences in air cavities because of different patient setups between MR and CT scans, the air volume considered only air voxels within the overlap of sCT and CT (less than −800 HU). ME and MAE of whole body voxels were calculated from the voxels within the overlap of external head contours of sCT and CT. The HU differences were calculated in corresponding voxels in sCT and CT.

## 3 Results

**Figure 2:**
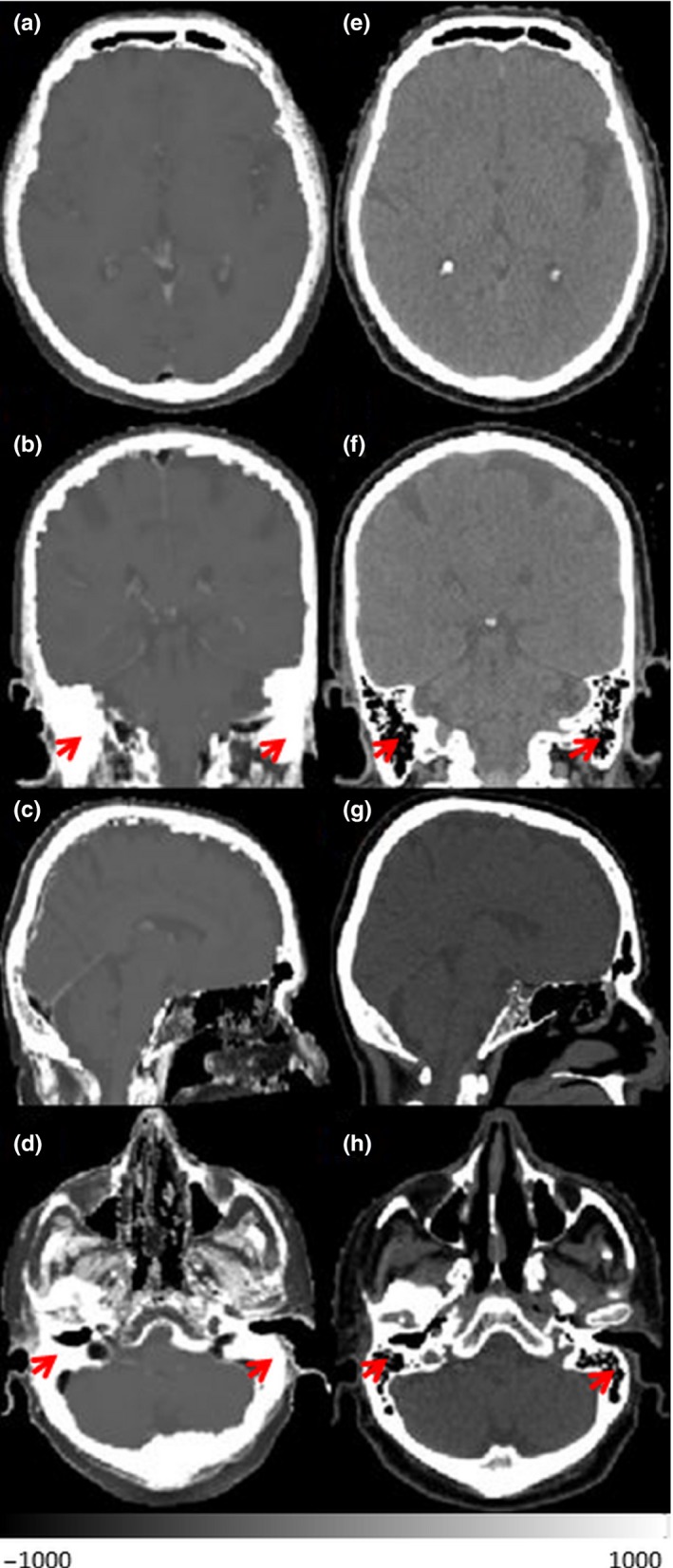
Synthetic‐CT (a–d) and corresponded CT (e–h) in three views and different axial level of one patient. Red arrows indicate mastoid sinuses

Figure 2 shows the sCT and CT in three views. sCT is visually similar to the CT. The main differences appear close to the edges of brain and CSF. The soft‐tissue contrast was preserved in sCT. Brain, CSF, brain ventricle, and brain stem can be clearly seen.

**Figure 3:**
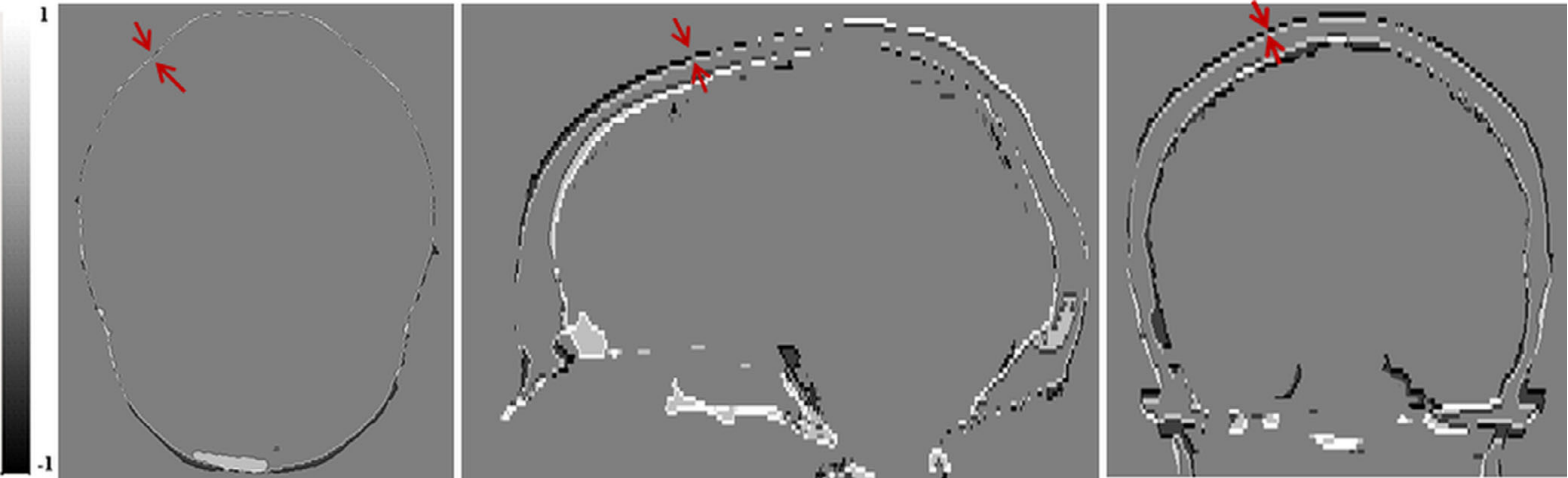
Differences of skull segmentation of synthetic‐computed tomography (sCT) and CT in Axial, Sagittal, and Coronal views. Red arrow indicated the distance between largest external contours of sCT and CT. Black is external skull edge (−1) from sCT and white (1) is external skull edge from CT. Gray is when the edges are overlapped (0).

Figure 3 shows the difference of the external contour of the skull in axial, coronal, and sagittal slices within the largest width of the skull. The averaged (and ±1SD) distances of maximum external contour of skull between sCT and CT are 0.6 ± 0. 3 mm, 1.3 ± 0.1 mm, 1.2 ± 0.2 mm in axial, coronal, and sagittal slices, respectively. The averaged distance in three views is 1.0 ± 0.1 mm.

**Figure 4:**
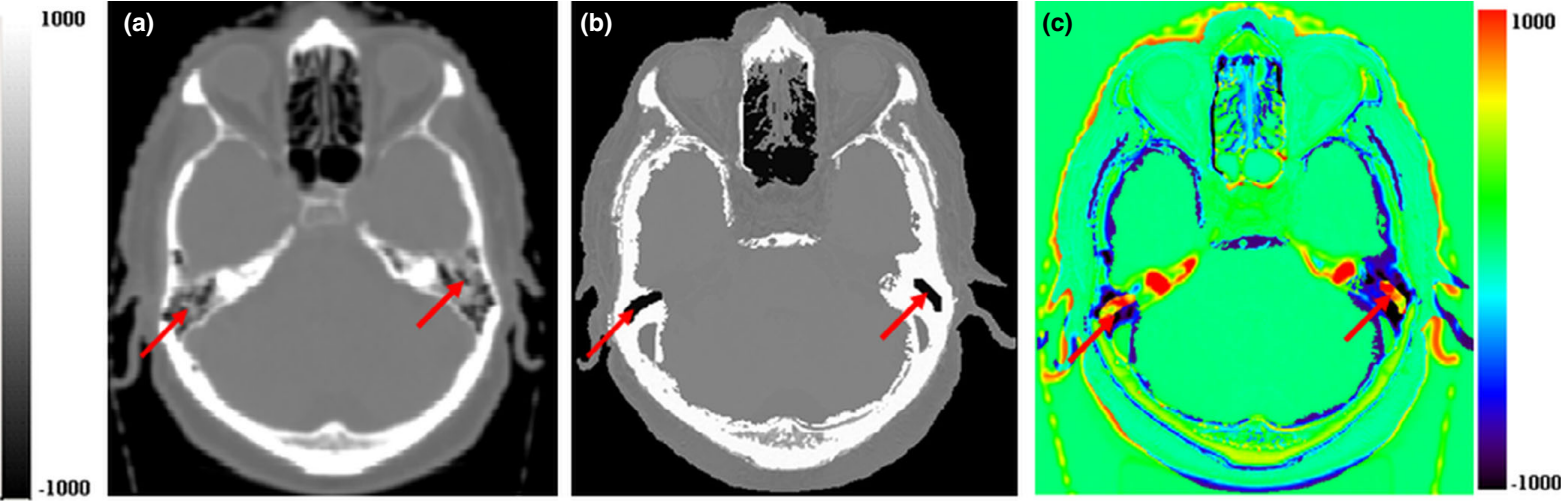
ED difference map between computed tomography (CT) and magnetic resonance. (a) CT slice, (b) synthetic‐CT slice, and (c) ED difference map between (a) and (b). Red arrows indicate mastoid sinuses

Figure 4 shows a HU difference map between sCT and CT. Large difference of soft tissue appears at part of the external contour of patient near the table top due to different shape of table tops (curved and flat) used in acquiring MR and CT scans. Significant differences can also be seen at mastoid sinuses which consist of small air bubbles separated by thin lamina bones.

The dice similarity coefficients for bone and brain (including CSF) are 0.72 (σ = 0.04) and 0.86(σ = 0.02), respectively. The ME and MAE for difference in soft tissues are shown in Table 2.

**Table 2:** The electron density error for tissues and DSC of bone and brain

Electron density error (CT/sCT) HU	Air	Brain	Bone	Muscle	FAT	Soft tissue (−100 to 200) HU	Whole‐body (external body contour)
MAE Avg. ±1SD Range	21 ± 2 (19–24)	8 ± 7	244 ± 18 (219–277)	23 ± 1	47 ± 5	26 ± 2 (22–30)	125 ± 9 (110–144)
ME Avg. ±1SD	−4 ± 3	1 ± 2	−34 ± 42	−5 ± 6	12 ± 16	2 ± 5	2 ± 15
DSC	–	0.86 ± 0.02	0.72 ± 0.04	–	–	–	–

CT, computed tomography; sCT, synthetic‐CT; DSC, dice similarity coefficients; MAE, mean absolute error; ME, mean error.

**Figure 5:**
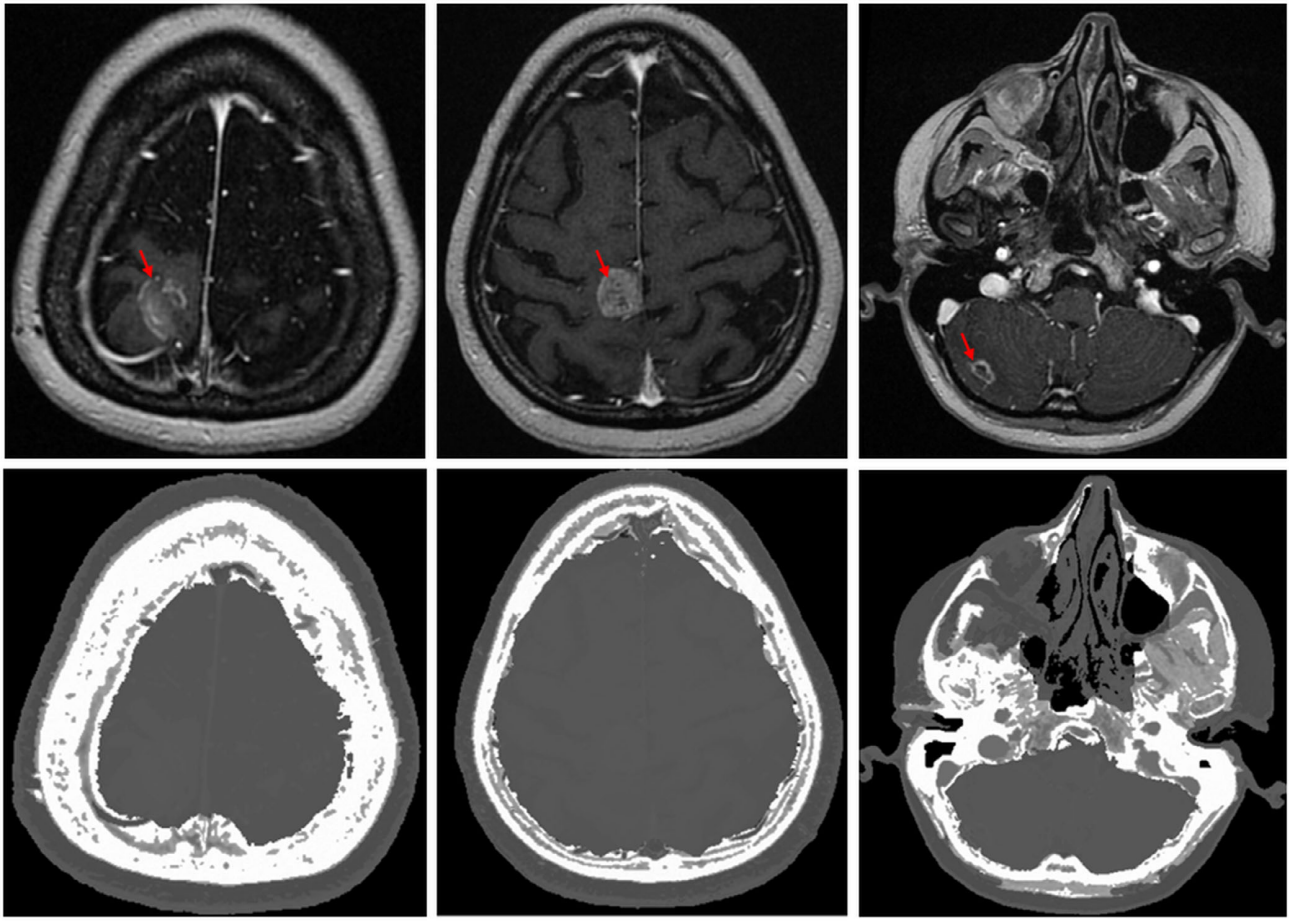
Magnetic resonance slices (upper row) and corresponding synthetic‐CT slices (lower row) for three patients. Left most images show a patient who has undergone surgery and leaky cerebrospinal fluid (left case) and enhanced tumors (red arrows)

Figure 5 shows sCTs generated three patients. Our method can consistently generate sCT for cases with significant pathological variation such as holes on the skull and tumors enhanced by contrast.

The average contouring time for air region was 10 min using contouring tools in Pinnacle^3^ treatment planning system and the average time for automated tissue segmentation and electron density mapping was about 2 min using an in‐house program, on a standard PC (CPU 2.6 GHZ, 8.0 GB RAM).

## 4 Discussions

In this work, we developed an electron density assignment method to generate sCT with one set of clinically used T1‐weighted MR images based on automated tissue segmentation techniques. The new method not only utilized information of boundaries among tissues through edge detection, it also used other information such as high tissue contrast, anatomical information (location, shape, and size), and tissue texture to differentiate tissues with similar intensities and assigned them corresponding voxel‐based electron densities.

The geometrical accuracy of generated sCT was evaluated by calculating average distances between external contours of the skulls of sCT and CT. The average difference was about1 mm. The largest difference was in the longitudinal direction. This may be due to the slice thickness of MR images which was 2.5 mm or 1.5 mm and was thicker than that 1.0 mm of CT. Different scan position was another factor. As shown in Fig. 3, the mismatching of skull contours of sCT and CT was mainly due to small differences in the positional angles between the neck and the base of the skull between MR and CT scans which cannot be corrected with rigid image registration.

As shown in Fig. 4, large ED errors were present in mastoid sinuses which are complex structures consisting of small air bubbles with thin bony separators. They were not always distinguishable from surrounding skull bone. Large ED assignment errors in these regions were also observed in other atlas, UTE, and CNN‐based techniques.^12^, ^14^, ^15^, ^16^ Due to the small volume of mastoid sinuses and the average effect of mixture of bone and air components, the impact of the ED errors on the overall accuracy of sCT is minimal. Errors also occurred near oral cavities because of different patient setup and at the neck where it is close to the edges of field of view (FOV) because of MR artifacts.

In our method, voxel‐based ED mapping was performed by assigning statistical ranges of CT numbers to voxels of individual tissues to minimize ED errors for each type of tissue so that the errors of overall ED assignment was minimized. Studies show that sCT generated using bulk ED assignment or voxel‐based ED assignment all achieved high overall dosimetric accuracy (<5%) compared with CT as ground truth.^22^, ^23^, ^24^, ^25^, ^26^, ^27^ The large dosimetric errors were found where targets and OARs were located near air cavities or bones when bulk ED assignment was used.^10^, ^26^ The ED of cortical bone can be as high as 2000 HU, and so the ED errors can be more than 1000 HU when 800 HU was used for bulk ED assignment of bone. Therefore, this method used voxel‐based approach to map EDs for cortical bone and trabecular bone. It achieved the average MAE of bone 244 HU which is significantly better than 422 HU reported in Ref. [9]. which used UTE sequence and bulk density assignment. It is worse than 130 HU of a study^7^ using multiple atlases. However, the large errors appear at regions which have large image registration errors such as near the oral cavities, base of skull near the neck due to different scan, and patient setups between MR and CT scans. The DSC of bone is 0.72 with is comparable with 0.73 in a study^14^ which utilized Zero TE (ZTE) sequence imaging.

Whole‐body MAE of our method is on average 126 HU which is better than 147 HU reported by a UTE study^10^ and is comparable to 123 HU from a ZTE study.^14^ Studies based on CNN and multiple atlas deformable registration in general reported lower whole‐body MAEs than the other methods. Hans et al. reported 85HU using a deep CNN method.^15^ Dinkla *et al*. and Farjarm *et al*.^7^, ^16^ reported 67 and 64 HU using CNN and multiple atlases techniques, respectively. However, it is difficult to compare different methods just based on whole‐body MAEs. The results of these CNN and multiple atlas methods^7^, ^16^ were achieved through cross validation using strictly selected “good” training data. The MR images were acquired in the clinical MR treatment protocol which has the same setup as that of CT, high resolution, thin slice thickness, identical MR sequence, and similar field of view. Cases with streaking artifacts on the CT and with noticeable image registration errors in air regions were excluded. Our study did not have specific exclusion criteria for validation data. We include all patient data acquired within a period of time as long as the field of view (FOV) includes the whole skull. Some patients even have streak dental artifacts on the CT.

One main advantage of our method compared to other methods is that it is robust with respect to patient‐related variations due to differences in anatomy, pathological conditions, and aging process. We have tested our method on a variety of patient cases in this study with different anatomies, including postsurgical patients and with contrast enhanced tumors as shown in Fig. 5. The results show that our method is robust to patient‐related anatomical and pathological variation. Both deep learning‐based CNN and multiple atlas‐based deformable registration methods have limitation on nonstandard cases with large anatomic and pathologic variation from training data or atlases. Some studies pointed out that these methods are not robust to patient‐related variation and suggested that increasing training data to include a variety of anatomy and pathological conditions may get around the issue but at the expense of processing time, dramatically increasing the required computing resources. Furthermore, using a large amount of training data can considerably increase the complexity of the trained kernel which poses a risk of skewing the kernel and making it prone to errors.

Another main advantage of our method compared to other methods is that it is robust to tissue intensity variation due to differences in scan protocols, scan parameters, and/or scanner type. Our method utilized prior knowledge of each type of tissues such as its location, relative position, tissue contrast, edges, shape and size to automatically search for the representative tissue voxels within each type of tissue region to learn the intensity variation of different tissues for each individual patient. Therefore, our method is not susceptible to scan‐related intensity variation acquired with heterogeneous scan protocols and parameters, and mitigates the impact of MR intensity variation due to nonuniformity of magnetic field and other image artifacts. We have tested the robustness of our method using validation MR images from two cancer programs which were acquired with different MR scan protocols, scan parameters, and scanners. Other atlas and deep learning‐based methods reduce the impact of MR scan‐related variation by strictly using same scan protocol, scan parameters, and high resolution to acquire and/or select “good” training data. The approach produced good results with the training data. But it limits the feasibility to apply these methods to other programs with heterogeneous data acquired using different MR scan protocols, scan parameters, and/or MR scanners.

Finally, the feasibility of our method is due to its simplicity to implement compared to multiple atlas‐based and deep learning‐based methods. Our method can be easily integrated into existing MR‐Sim programs using their existing MR scan parameters, MR scanners, and computing resources without significant costs. Our method can be easily validated using existing MR data without the need to gather a large amount of homogeneous training data which is only feasible for a few large programs. On the contrary, other methods need a high‐resolution scan protocol in 3D which will dramatically increase scanning time. The tremendous computing complexity of these methods requires computers with GPUs for training and generating sCTs. This would put a great burden to existing treatment planning and information record and verification system and would be associated with significant costs.

One limitation of this method is that it needs manual contouring nasal cavities and airway regions. It took on average of about 10 min in this study. However, our approach is less time consuming than other methods in running time considering the long time from 2 to 12 h for multiple atlas image registration [6.9], and long training time of the kernel for CNN approach which can take days^15^, ^16^ and additional MR scan time for UTE approach. Although some studies can reduce the processing time to a few minutes by using GPUs and large RAM,^15^, ^16^ our method is still comparable in terms of cost of effective and simplicity of implementation. The manual contouring time can be further reduced by improving our air segmentation algorithm so that the manual contouring can be reduced to just drawing an air sample box in sinuses or potentially be eliminated with an automating air segmentation step.

The study did not evaluate DSC of air segmentation since it is difficult to compare with those of CT for our dataset. Significant differences of nasal cavities and airway between registered MR and CT images were noticed in our dataset as the MR and CT scans were taken in different position, setup, and different time. Unlike for the rigid skull, the volume and position of nasal canal and airway can be significantly varied with scan position and setup.

The novelty of our approach is that our method generated a sCT directly from one set of clinical T1 MR images. It does not require multiple atlas‐based deformable image registration. Therefore, it eliminates image registration error. The method does not require slow training with a large number of training data as CNN approaches. The method does not need special MR scans for bone. Therefore, it does not increase any cost by utilizing T1‐weighted MR images from current SRS or SABR protocols which have high geometric precision and a good quality control. It fits well with the newly developed MR guided radiotherapy (MRGRT) treatment devices such as Elekta MR‐Linc and ViewRay MRIdian. Our method can be extended to automatically generate contours of organs of risk in the head which is an advantage for MR‐only RTP. The work flow with our method is quite simple to implement and more economic than the current approaches.

In future work, sCTs generated by this method will be evaluated dosimetrically using SRS and SABR patient data from multiple centers. T1‐weighted MR images of these patients are acquired using different parameters, scanners, and protocols. The feasibility of using sCT to substitute CT for patient setup verification by registering with cone beam CT will be evaluated.

## 5 Conclusion

A new MR electron density mapping technique was developed based on tissue segmentation information from only one set of clinical T1‐weighted images with contrast. The generated synthetic‐CT is comparable to that of CT in terms of anatomical position of tissues and similarity of ED assignment. This method is a practical method for MR‐only radiotherapy. It applies to a variety of patient anatomy and is robust to MR intensity variation.

## Conflict of Interest

No conflicts of interest**.**
